# Observation of Contrary Thermo-responsive Trend for Single Crystal and Powder Samples in Mechano-, Thermo- and Solvato-responsive Luminescent Cubane [Ag_4_I_4_L_4_] Cluster

**DOI:** 10.1038/s41598-017-11974-8

**Published:** 2017-10-12

**Authors:** Shi-Li Li, Min Han, Bin Wu, Jie Wang, Fu-Qiang Zhang, Xian-Ming Zhang

**Affiliations:** 1 Key Laboratory of Magnetic Molecules & Magnetic Information Materials (Ministry of Education), School of Chemistry & Material Science, Shanxi Normal University, Linfen, Shanxi 041004 P. R. China; 2 Institute of Crystalline Materials, Shanxi University, Taiyuan, Shanxi 030006 P. R. China

## Abstract

A new silver(I) iodide cluster [Ag_4_I_4_(TMP)_4_] (TMP = tris(3-methylphenyl)-phosphine) **1** shows triply stimuli-responsive luminescent chromism, namely, mechano-, thermo- and solvent-responsive chromism, which is isostructural to our previously reported [Cu_4_I_4_(TMP)_4_] **2** but shows quite different luminescence in response to the external stimuli. Especially, during the mechanical grinding, the relative intensities of HE and LE of **1** varied with a concomitant hypsochromic shift, and when the temperature was decreased from 300 to 5 K, unprecedented contrary thermo-responsive trend for single crystal and powered samples (blueshift of single crystals and redshift of powdered samples) was observed. These distinct characters of **1** should be due to the different molecular packing modes, metallic interactions and the unique character of Ag(I) ion.

## Introduction

Phosphorescent coordination materials have received increasing attention due to their fascinating functionalities and widespread applications in lighting, sensing, memories and display devices, which provides a great motivation for research on coinage metal emissive complexes^1–5^. As an important member of these materials, coinage metal emissive complexes, such as coinage metal-halide aggregates, exhibit modification of their luminescent color upon exposure to the external stimuli (for example, temperature, mechanical grinding, light, pH, solvent vapor, etc)^6–10^. Such functional compounds reported so far are mainly focused on copper-halide-based complexes due to their chromic luminescent properties which are generally related to the flexible geometries. The majority of known copper-halide-based complexes display stimuli-responsive chromic luminescent behaviors; however, the emission shifts of these compounds are unpredictable, and the origin of stimuli-responsive chromisms, particularly mechanochromic luminescence, remains ambiguous^11,12^. Stimuli-responsive chromisms, except thermochromism, of copper halide complexes have not been well investigated yet because structural changes in response to the external stimuli cannot be comprehensively determined through single-crystal X-ray crystallography or other experimental methods^13^. Especially, during mechanical grinding, the crystal state of copper cluster complexes is generally converted into the amorphous state, which is difficult to characterize through single-crystal X-ray diffraction analysis. Scholars have proposed different hypothetical interpertation of the origin of various chromisms, but no general rule has been established. Studies on the number of stimuli-sensitive luminescent complexes still remain in the infancy stage; thus, novel stimuli-responsive chromic luminescent complexes must be developed to comprehensively investigate chromic properties and provide new insights into their origins.

Similar to the Cu(I) ions of the stimuli-responsive copper(I) halide clusters, Ag(I) ions possess the tetrahedral coordination geometry and exhibit metallophilic interactions; hence, emissive silver(I) halide compounds are potential stimuli-sensitive materials. In addition, the coordinated geometry of Ag(I) ion can change from a tetrahedral geometry to a square-planar geometry under an external stimulus; this property is similar to that of copper-halide clusters and can induce the striking tunable luminescence^14^. Nevertheless, some differences exist between the copper(I) halide and silver(I) halide compounds. (1) Ag(I) ion is more inert to oxidation than Cu(I), often resulting in an absence of the low-energy MLCT state and reduced complexity of the origin of stimuli-responsive luminescence^15^; (2) In contrast to Cu(I) complexes, the geometrical arrangements and local environment around Ag(I) ions drastically affect luminescence^16^. Considering these similarities and differences, scholars have focused on stimuli-responsive luminescence of emissive silver(I)-halide materials^17^. Nevertheless, research on emissive silver(I) halides is extremely limited because of their potential photosensitivity and low luminescent efficiency^18^. In particular, the luminescent properties of tetranuclear Ag(I) clusters formulated [Ag_4_I_4_L_4_] (L = organic ligand) have been rarely investigated; however, a number of isostructural tetracopper(I) complexes have been intensively explored for their luminescent stimuli-responsive properties^19–21^. Further studies on multinuclear silver(I)-halide materials must be conducted because of their interesting emission properties and distinct emissive mechanisms. Based on above considerations, we aim to design and synthesize new silver(I) cluster-based compound with stimuli-responsive luminescence by using hydro/solvothermal synthesis method. Herein, we develop new cubane [Ag_4_I_4_(TMP)_4_] complex (**1**) with weak Ag-Ag interaction; this complex is isostructural to the compound [Cu_4_I_4_(TMP)_4_] **2** in the absence of Cu-Cu interaction, as previously reported by our team^12^. However, compound **1** exhibits quite different luminescence in response to external stimuli. In particular, an unprecedented contrary thermo-responsive trend was observed for single crystal and powder samples (blueshift of single crystals and redshift of powdered samples with decreasing temperature). Compound **1** displayed distinct chromic luminescence probably because of its closed packing mode, presence of metallic interactions, and unique characteristic of Ag(I) ions. These results contribute to understanding the effects of the metallophilic interactions, molecular structure, and packing mode for stimuli-responsive luminescence.

## Results

### Description of Structures

**Figure 1:**
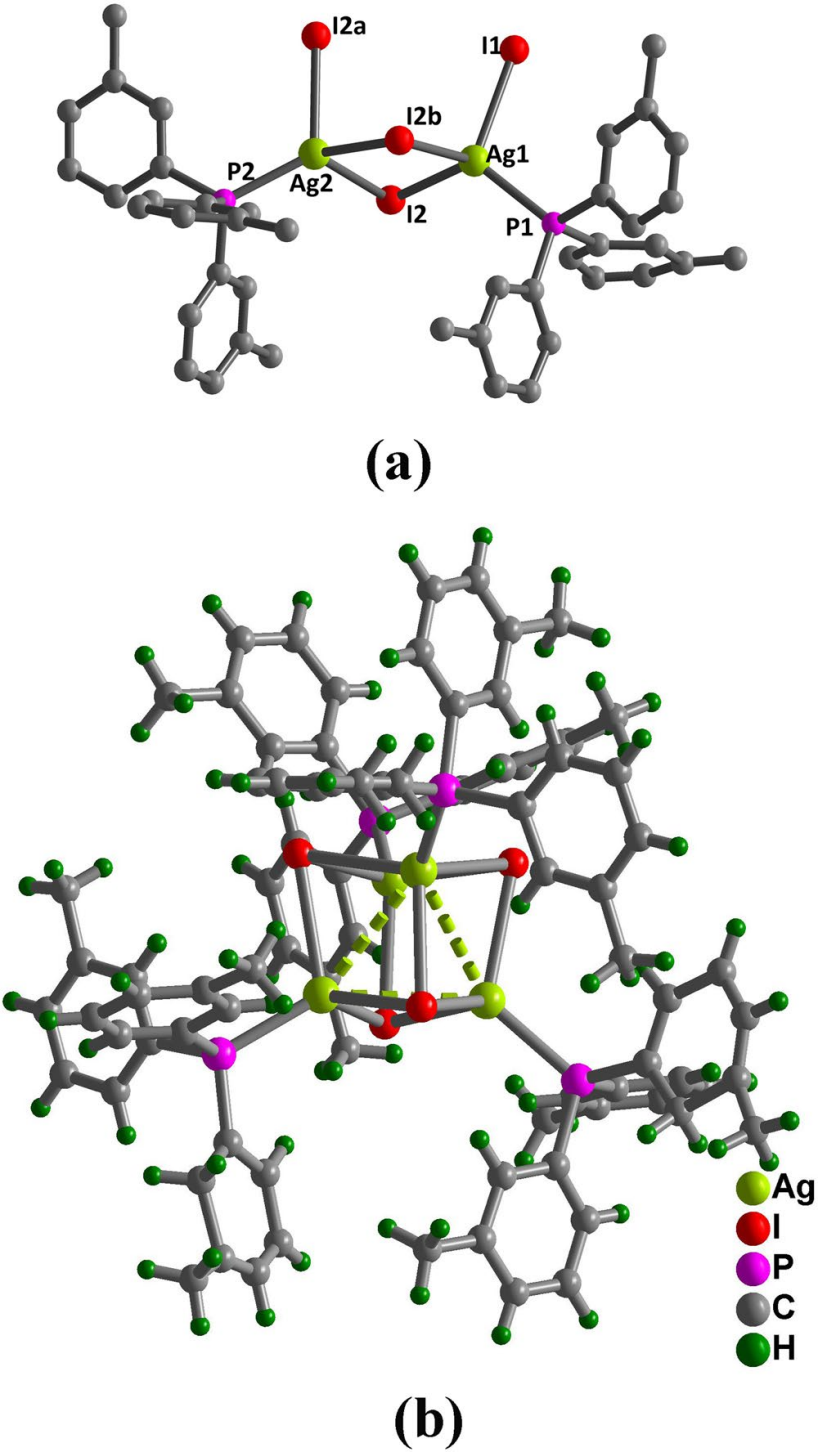
View of the coordination environment of Ag(I) ions (**a**) and molecular structure (**b**) in **1**. Selected bond lengths (Å) and angles (°). At 293 K: Ag(1)-P(1), 2.460(3); Ag(1)-I(1), 2.9271(12); Ag(1)-I(2a), 2.9122(11); Ag(1)-I(2), 2.8782(11); Ag(2)-P(2), 2.450(4); Ag(2)-I(2), 2.9310(9); Ag(2)-I(2a), 2.9311(9); Ag(2)-I(2b), 2.9311(9); Ag(1)-Ag(1a), 3.3762(15); Ag(1)-Ag(1b), 3.3764(15).

X-ray diffraction data reveals that compound **1** contains cubane [Ag_4_I_4_] clusters with four silver ions and four iodide ions alternatively occupying the corners of a distorted cube. Compound **1** with chiral *C*_3_-symmetric cubane structure crystallizes in cubic space group I-43*d*. The asymmetric unit contains one third of a cubic [Ag_4_I_4_] core located on the crystallographic threefold axis; in the axis, two crystallographically independent Ag atoms adopt a distorted AgPI_3_ tetrahedral geometry and are coordinated three *μ*_3_-iodide ions and one phosphorus donors from the TMP ligand (Fig. 1). The Ag-P bond lengths of 2.460(3) and 2.450(4) Å at 293 K are comparable with those of the related clusters^22–24^. The Ag-I bond distances are within the range of 2.8782(11) and 2.9311(9) Å, and the I-Ag-I bond angles range from 96.83(3) and 107.74(3) °. The Ag-Ag distances within the range of 3.3762(15) and 3.3764(15) Å at 293 K are shorter than the sum of the van der Waals radii (3.44 Å), indicating the presence of argentophilic interaction in compound **1** ^17,25^. In the isostructural [Cu_4_I_4_(TMP)_4_] **2**, the metallophilic interaction is absent at room temperature^12^. Intermolecular offset π···π interactions exist between two phenyl rings of neighboring [Ag_4_I_4_(TMP)_4_] clusters. The centroid-to-centroid distance between two closest benzene rings is 3.900(8) Å and the corresponding dihedral angle is 2.82 ° (Table S2 and Figure S1).

### Photoluminescence Properties

**Figure 2:**
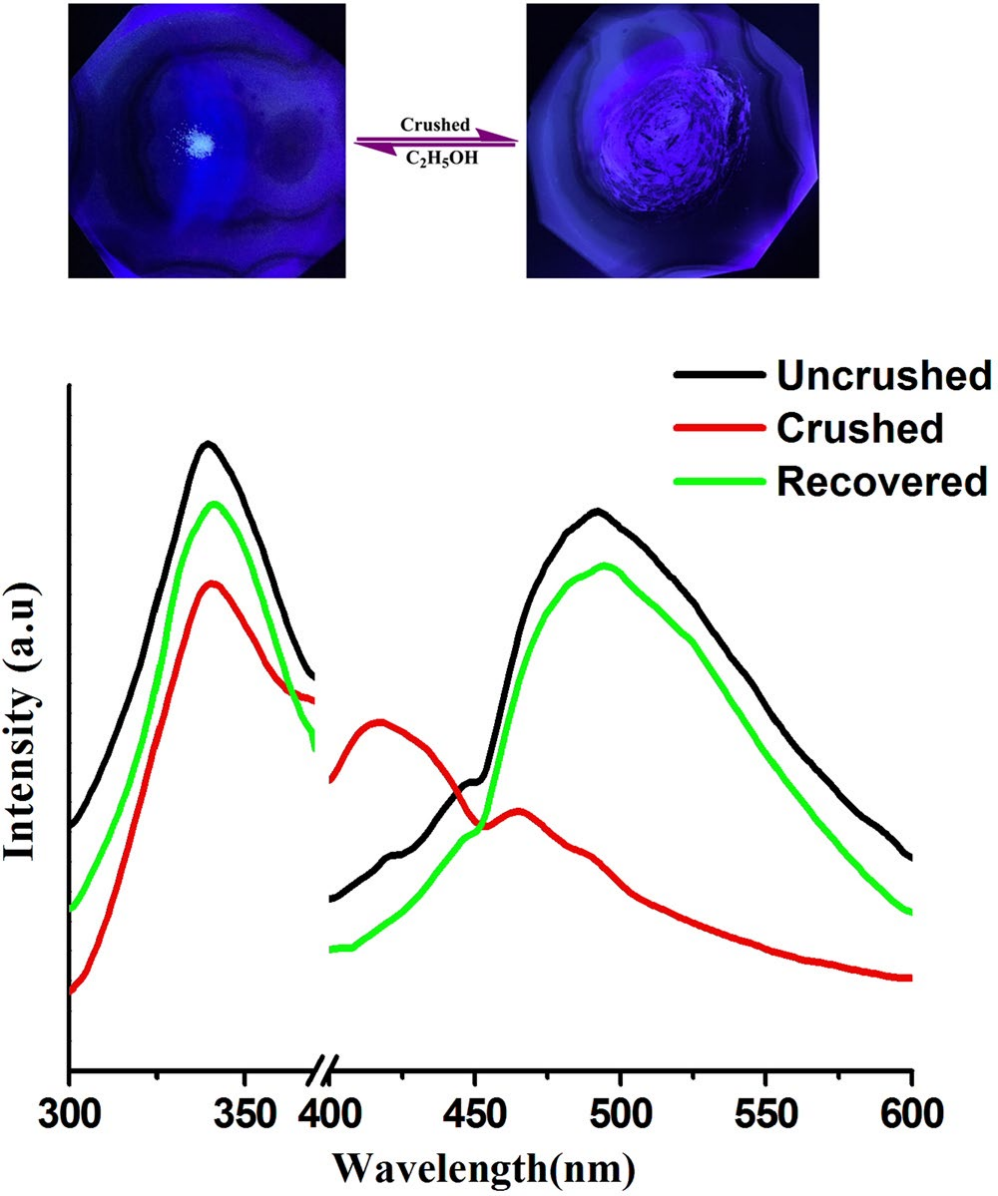
The luminescence spectra of compound **1** at room temperature in different states.

The photoluminescent spectra of **1** were measured in depth and reveal triply stimuli-responsive luminescent chromism, namely, mechano-, thermo- and solvato-responsive chromism. A detailed photophysical study of the single-crystal **1** shows a weak high-energy emission (HE) centered at 448 nm (*τ* = 0.8 *μ*s) and a strong low-energy emission (LE) centered at 476 nm (*τ* = 1.3 *μ*s) under excitation at 337 nm. With carefully reviewing the relative literatures, the HE and LE emission bands can be generally clarified as follow. The HE emission band is probably induced by the intraligand π-π* charge transitions (ILCT) of the TMP ligands^25–27^. And the LE emission band can be assigned to the halide-to-ligand charge-transfer (XLCT) transition with a minor contribution of metal-ligand charge transfer (MLCT)^13,18,28^. Compared with that of **2**, the emission peaks of **1** exhibit an evident blue shift, which is due to the lower d-orbitals of Ag(I)^29^. The internal absolute quantum yield for **1** is 7% at room temperature after excitation at 337 nm. When the single crystal of compound **1** was crushed using a pestle in an agate mortar, the luminescence colour was gradually converted from blue-green into blue-purple and the corresponding intensity decreased (Fig. 2). Compared with the single-crystal sample, the emission peaks of ground powder exhibits a slight blue shift; the spectra indicate intense HE peak at 418 nm with *τ* = 0.4 *μ*s and weak LE peak at 468 nm with *τ* = 0.6 *μ*s. To be noted, after grinding, the HE band became much stronger than LE band, which is not observed in **2** and other mechanochromic luminescent cubic Cu_4_I_4_ clusters^12,30^. To our best knowledge, this phenomenon is only observed in rare coinage-metal halide coordination polymer and organic polymers and could be due to the structural deformation and variation in intermolecular or intramolecular interaction induced by mechanical stimuli^25,31,32^. The extraordinary mechanochromic luminescence might be attributed to the rise in energy levels of the lowest unoccupied molecular orbitals (LUMOs) caused by alterations in weak interactions, such as Ag···Ag and π···π interactions^11,33^. The shortened lifetime, decreased quantum yield, and weakened intensity of the powdered samples might be due to the reduced crystallinity of **1** during grinding, as reflected in PXRD diagrams^34,35^. Consequently, complex **1** displayed unusual mechanochromic properties with tunable relative emission intensities of LE and HE bands and blue-shifting in response to mechanical stimuli. In contrast, the mechanochromic luminescent cubane Cu_4_I_4_-cluster-based compounds, the emission bands in the grinding process usually shift to longer wavelength with the concomitant increasing of emission intensity, lifetime and internal luminescence quantum yield^13,36^. The Raman spectra of the ground and unground samples are almost the same and do not reveal a new signal, indicating the lack of apparent phase transition in the grinding process (Figure S2). The PXRD patterns of the ground and unground samples are consistent with those calculated from the single-crystal X-ray structure, implying that the mechanochromic luminescence is not concomitant with phase transition (Figure S3). Generally, the minimal difference of PXRD patterns between the ground and unground samples demonstrate the presence of slight structural changes after grinding. Upon dropping ethanol to the powder sample and subsequent evaporating of ethanol, emission color slowly returned to the original blue-green.

**Figure 3:**
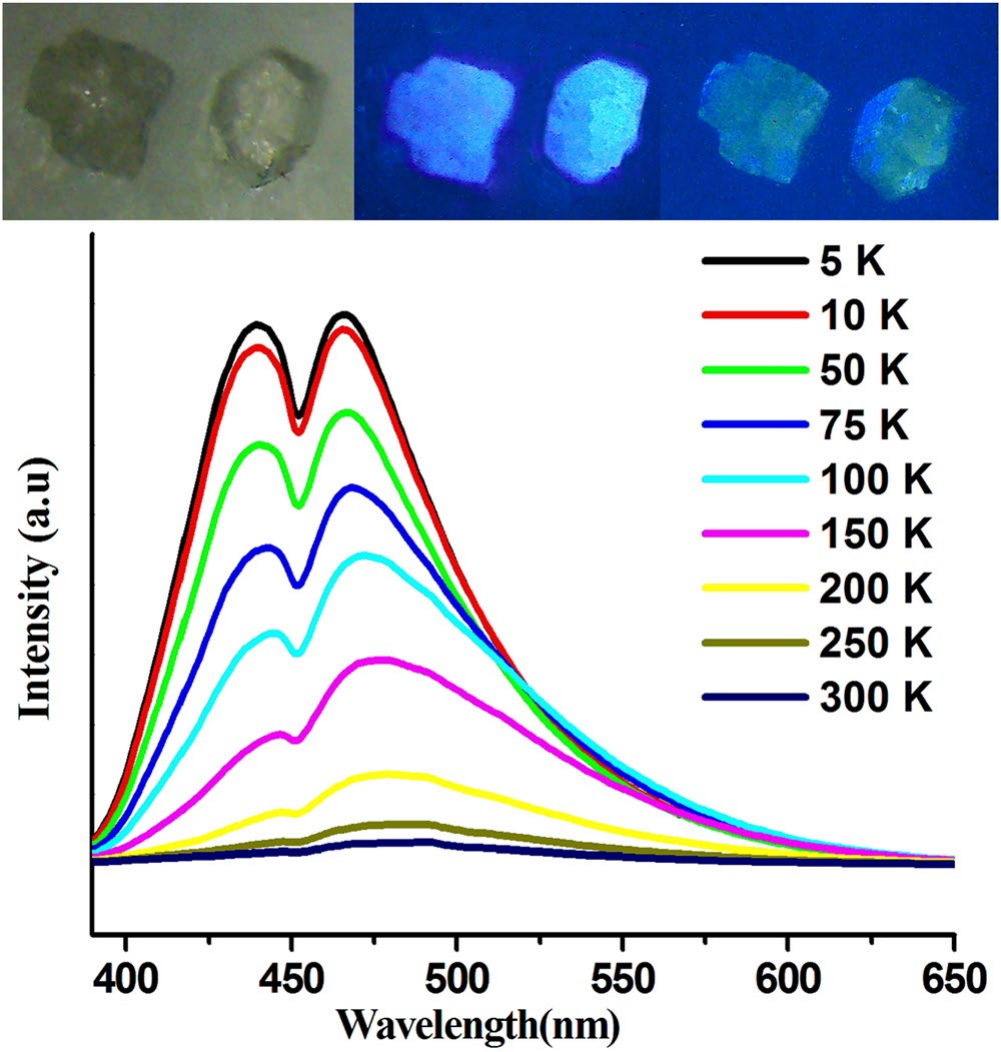
Temperature-dependent emission spectra of single crystal **1** from 5 to 300 K. Inset: Images for single crystals under ambient light at room temperature (left), under 365 nm UV lamp irradiation at room temperature (middle) and in liquid nitrogen (77k) (right).

**Figure 4:**
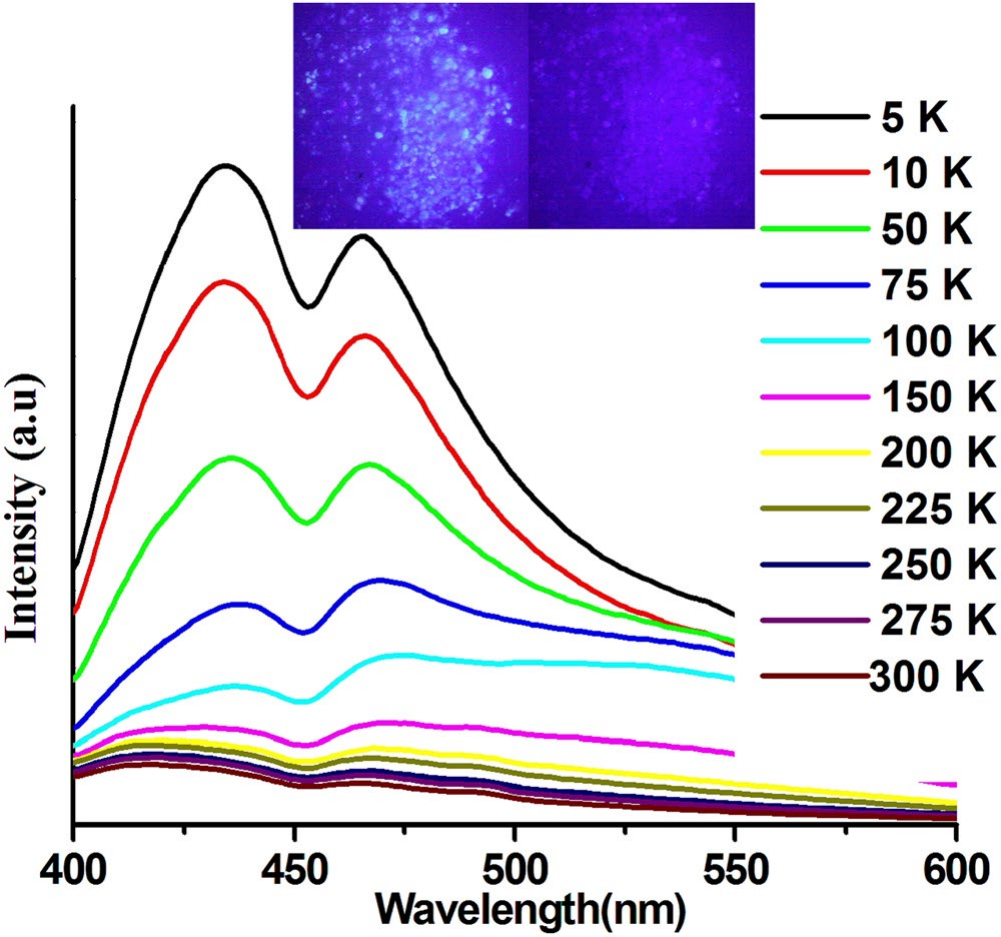
Temperature-dependent emission spectra of **1** in ground powder state from 5 to 300 K. Inset: Images for powder sample under 365 nm UV lamp irradiation in liquid nitrogen (left) and at room temperature (right).

The thermo-responsive luminescent chromism of the solid-state compound **1** was also studied in detail. A unique contrary thermo-responsive trend was found for both single and powdered sample. After immersion in liquid nitrogen for a few minutes, both unground and ground samples were collected and exposed to the UV light. The reversible thermochromic luminescence for both samples can be easily distinguished by the naked eye (Figs 3 and 4). For single-crystal sample, the LE emission band progressively blue-shifted to 466 nm and the HE emission band slightly shifted to 440 nm with decreasing the temperature to 5 K. Such blue-shifted emission observed with decreasing temperature is very rare in coinage-metal thermochromic luminescent coordination compounds^37^. This unusual phenomenon is very complicated and possibly related to variations in synergistic π···π and Ag···Ag interactions with decreasing the temperature. The intensities of both LE and HE emissions gradually increased with decreasing temperature, but the intensity of HE band increased faster than that of LE band. When the unground single-crystal sample was cooled down to 5 K, the intensity of the HE band is almost equal to that of the LE band (Fig. 3). The measured lifetimes were increased drastically by approximately 100 times from 0.8 *μ*s at 300 K to 97.4 *μ*s at 5 K for HE band and from 1.3 *μ*s at 300 K to 90.5 *μ*s at 5 K for LE band, respectively (Table S3 and Figures S5–13). The emission lifetimes significantly increased with decreasing the temperature. This finding could be due to the relation of decreased non-radiative rate to the high spin-orbit coupling of Ag(I) ions and fast intersystem crossing^38^. This behavior is also observed in the emissive copper(I) and silver(I) complexes with the thermally activated delayed fluorescence^39,40^.

The thermochromic luminescence properties of compound **1** were also affected by the mechanical grinding. After crushing, the luminescence of the powdered sample **1** exhibited unprecedented contrasting thermochromic luminescence trend from that of single-crystal **1**. With decreasing temperature, the HE emission band of the powder sample progressively increased and red shifted to 435 nm with a concomitant increase and slight red shift of the LE band, which is contrary to the blue-shifted emission observed in the single crystal sample. To be noted, two different changing steps in the thermochromic luminescent process of powder sample were observed in the solid-state emission spectra (Figure S14 and S15). Initially, the intensity of LE emission band increased faster than that of HE emission band from room temperature down to 150 K until the intensities of the LE and HE bands are almost equal. While with continuous decrease in temperature to 5 K, the intensity of the HE emission band increased faster; at 5 K, the HE intensity is much higher than LE intensity (Fig. 4). When the temperature was lowered to 5 K, the lifetimes largely increased by hundreds of times from 0.4 *μ*s at 300 K to 168.5 *μ*s at 5 K for HE band and from 0.6 *μ*s at 300 K to 175.6 *μ*s at 5 K for LE band, respectively (Table S4 and Figure S16–24). It can be seen that the luminescent lifetimes of powder samples increased much faster than that of single crystal sample.

Solvato-stimuli experiments were also carried out. The crushed powder sample of **1** was immersed into six different organic solvents, ultrasonicated, and then aged for 48 hours to form the stable emulsion solutions. The luminescent spectra in most solvents such as EtOH, BuOH, DMF, acetone, and cyclohexane displayed obvious changes. The luminescent emission bands became nearly the same as that of unground sample, indicating that these solvents have effect to mediate the recovery of powder sample to original structural arrangement^41,42^. While in CH_3_CN solvent, the emission bands showed no changes (Figure S25). In addition, we carefully performed vapor-stimuli experiments. When the colorless block crystals of **1** were exposed to CH_3_CN, CH_2_Cl_2_, C_2_H_5_OH, BuOH and DMF vapors respectively, the luminescent color and emission bands remained unchanged, indicating none vapochromism.

## Discussion

The developed new cubic silver(I)-iodide cluster exhibits mechano-, thermo- and solvato-responsive luminescent chromism. Although this compound is isostructural to our reported copper(I)-halide compound, their multistimuli-responsive luminescence differ upon exposure to the external stimuli. In particular, after mechanical grinding, the relative intensities of HE and LE of **1** varied with a concomitant hypsochromic shift, when the temperature was decreased from 300 to 5 K, an unprecedented contrary thermo-responsive trend was observed for single crystal and powder samples (blueshift of single crystals and redshift of powdered samples with decrease of temperature). The chromic luminescence of **1** is considerably different from those of **2** and other chromic luminescent cubic Cu_4_I_4_ clusters. These distinct characters of **1** might be due to the different molecular packing modes, metallic interactions and the unique character of Ag(I) ion.

## Methods

### Testing Methods

All reagents and solvents were obtained commercially and used without further purification. The FT-IR spectra were recorded from KBr pellets in range 400–4000 cm^–1^ on a Perkin-Elmer Spectrum BX FT-IR spectrometer. Elemental analysis was performed on a Vario EL-II elemental analyzer. XRPD data were recorded in a Bruker D8 ADVANCE X-ray powder diffractometer (Cu_Kα_, λ = 1.5418 Å).

Steady-state photoluminescence spectra and lifetime measurements were measured from 300 K down to 5 K by a single-photon counting spectrometer using an Edinburgh FLS920 spectrometer equipped with a continuous Xe-900 xenon lamp, and a μF900 microsecond flash lamp. The corrections of excitation and emission for the detector response were performed ranging from 200 to 900 nm. Temperature-dependent measurements were carried out in a JANIS SHI-4S-1 cold head cooled with HC-4A compression engine. The data were analyzed by iterative convolution of the luminescence decay profile with the instrument response function using the software package provided by Edinburgh Instruments. Lifetime data were fitted with triple-exponential-decay functions. The quantum yields were measured by use of an integrating sphere with Edinburgh Instrument FLS920 spectrometer.

### Synthesis

Synthesis of Ag_4_I_4_(TMP)_4_(**1**). A mixture of AgI (0.5 mmol, 0.152 g), KI (1 mmol, 0.165 g), TMP (0.2 mmol, 0.059 g) and CH_3_CN solvent (5 mL) was stirred and then sealed in a 25-ml Teflon-lined stainless container and heated to 140 °C for 7 days. With a cooling rate of 5 °C min^−1^ to room temperature, clourless block crystals of **1** in 56% yield were recovered. Anal. Calc. for C_84_H_87_Ag_4_I_4_P_4_ **1**: C, 46.72; H, 4.06; N, 9.47. Found: C, 46.65; H, 4.15; N, 9.53. IR(KBr, cm^−1^): 3440 s, 3118 s, 2906w, 2361w, 1603m, 1481 m, 1403 m, 1280w, 1102 s, 980w, 890w, 780 m, 679 m, 612w, 545w, 456w.

### Single Crystal X-ray Crystallography

X-ray single-crystal diffraction data for **1** at 293 K were performed with Mo-Kα radiation (λ = 0.71073 Å) on a Bruker Apex CCD diffractometer at 298(2) K. The program SAINT was used for integration of diffraction profiles, and the program SADABS was used for absorption correction. The structure was solved with XS structure solution program by direct method and refined by full-matrix least-squares technique using Olex2. All non-hydrogen atoms were refined with anisotropic thermal parameters. Hydrogen atoms of organic ligands were generated theoretically onto the specific carbon atoms, and refined isotropically with fixed thermal factors. Further details regarding the structural analysis are summarized in Table 1 and selected bond lengths and bond angles are shown in Table S1.

**Table 1:** Crystal data and structure refinement for complexes **1**.

Complex	**1**
Empirical formula	C_84_H_87_Ag_4_I_4_P_4_
Formula weight	2159.49
Temperature	293(2) K
Crystal system	Cubic
Space group	*I*-43*d*
*a* (Å)	32.2143(7)
*b* (Å)	32.2143(7)
*c* (Å)	32.2143(7)
*α* (°)	90
*β* (°)	90
*γ* (°)	90
*V* (Å^3^)	33431(2)
*Z*	16
*ρ*_calc_, (g cm^−3^)	1.716
*μ*, (mm^−1^)	2.518
F(000)	16816.0
Size (mm)	0.26 × 0.18 × 0.1
*θ*(°)	1.548 to 26.359
Reflections/unique	90321/5704
T_max_/T_min_	0.7868/0.5605
Data/parameters	5704/6/290
S	1.031
R_1_^a^, wR_2_^b^[*I* > 2*σ*(*I*)]	0.0358, 0.0835
R_1_^a^, wR_2_^b^(all data)	0.0532, 0.0958
∆ρmax/∆ρmin(eǺ^–3^)	0.64/−0.42

^a^ *R*_1_ = *F*_o_−*F*_c_/*F*_o_, ^b^ *wR*_2_ = [*w*(*F*_o_^2^−*F*_c_^2^)^2^/*w*(*F*_o_^2^)^2^]^1/2^.

### Solvatochromism Experiment section

Ground powder of **1** was immersed into various pure organic solvents such as DMF, acetone, cyclohexane, EtOH, MeCN, n-Butanol. After carried out 20 min ultrasound, the corresponding suspensions were obtained for measurements.

## Electronic supplementary material

Supporting Information

the Crystallographic Information Framework

the checkCIF PDF

## References

[1] Yam VW-W, Au VK-M, Leung SY-L (2015). Light-Emitting Self-Assembled Materials Based on d^8^ and d^10^ Transition Metal Complexes. Chem. Rev..

[2] Lu JY (2003). Crystal engineering of Cu-containing metal–organic coordination polymers under hydrothermal conditions. Coord. Chem. Rev..

[3] Peng R, Li M, Li D (2010). Copper(I) halides: A versatile family in coordination chemistry and crystal engineering. Coord. Chem. Rev..

[4] Vitale M, Ford PC (2001). Luminescent mixed ligand copper(I) clusters (CuI)_n_(L)_m_(L = pyridine, piperidine): thermodynamic control of molecular and supramolecular species. Coord. Chem. Rev..

[5] Fan GL, Yan DP (2014). Positional isomers of cyanostilbene: two-component molecular assembly and multiple-stimuli responsive luminescence. Sci. Rep..

[6] Wen T (2014). Redox-active Cu(I) boron imidazolate framework for mechanochromic and catalytic applications. Chem. Commun.

[7] Li B (2014). Thermochromic Luminescent Nest-Like Silver Thiolate Cluster. Chem. Eur. J..

[8] Zhang X, Chi Z, Zhang Y, Liu S, Xu J (2013). Recent advances in mechanochromic luminescent metal complexes. J. Mater. Chem. C.

[9] Zhan S-Z, Li M, Ng SW, Li D (2013). Luminescent Metal–Organic Frameworks (MOFs) as a Chemopalette: Tuning the Thermochromic Behavior of Dual-Emissive Phosphorescence by Adjusting the Supramolecular Microenvironments. Chem. Eur. J..

[10] Sun D (2013). Two birds with one stone: anion templated ball-shaped Ag_56_ and disc-like Ag_20_ clusters. Dalton Trans..

[11] Deshmukh MS, Yadav A, Pant R, Boomishankar R (2015). Thermochromic and Mechanochromic Luminescence Umpolung in Isostructural Metal–Organic Frameworks Based on Cu6I6 Clusters. Inorg. Chem..

[12] Yang K, Li S-L, Zhang F-Q, Zhang X-M (2016). Simultaneous Luminescent Thermochromism, Vapochromism, Solvatochromism, and Mechanochromism in a C_3_-Symmetric Cubane [Cu_4_I_4_P_4_] Cluster without Cu–Cu Interaction. Inorg. Chem..

[13] Benito Q (2014). Polymorphic Copper Iodide Clusters: Insights into the Mechanochromic Luminescence Properties. J. Am. Chem. Soc..

[14] Osawa, M. & Hoshino, M., Photochemistry and photophysics of the tetrahedral silver(I) complex with diphosphine ligands: [Ag(dppb)_2_]PF_6_ (dppb = 1, 2-bis[diphenylphosphino] benzene). *Chem. Commun*. 6384–6386 (2008). 10.1039/b813844c 19048163

[15] Matsumoto K, Shindo T, Mukasa N, Tsukuda T, Tsubomura T (2010). Luminescent Mononuclear Ag(I)−Bis(diphosphine) Complexes: Correlation between the Photophysics and the Structures of Mononuclear Ag(I)−Bis(diphosphine) Complexes. Inorg. Chem..

[16] Barbieri, A., Accorsi, G. & Armaroli, N. Luminescent complexes beyond the platinum group: the d^10^ avenue. *Chem. Commun*. 2185–2193 (2008). 10.1039/b716650h 18463736

[17] Li H-H (2006). Role of Spacers and Substituents in the Self-Assembly Process:  Syntheses and Characterization of Three Novel Silver(I)/Iodine Polymers. Cryst. Growth Des..

[18] Chen J (2016). Highly Efficient Thermally Activated Delayed Fluorescence in Dinuclear Ag(I) Complexes with a Bis-Bidentate Tetraphosphane Bridging Ligand. Inorg. Chem..

[19] Benito Q (2015). Mechanochromic Luminescence of Copper Iodide Clusters. Chem. Eur. J..

[20] Zhao C-W (2016). An *in situ* self-assembled Cu_4_I_4_-MOF-based mixed matrix membrane: a highly sensitive and selective naked-eye sensor for gaseous HCl. Chem. Commun..

[21] Zeng G (2016). 3d–4f Metal–Organic Framework with Dual Luminescent Centers That Efficiently Discriminates the Isomer and Homologues of Small Organic Molecules. Inorg. Chem..

[22] Teo B-K, Calabrese JC (1975). Synthesis, characterization, and bonding of tetrameric triphenylphosphine silver halide cluster systems. Evidence of dictation of stereochemistries by van der Waals interactions. J. Am. Chem. Soc..

[23] Bowmaker, G. A. *et al*. Spectroscopic and structural studies on 1:1 adducts of silver(I) salts with tricyclohexylphosphine*. J. Chem. Soc. Dalton Trans*. 2459–2465 (1996).

[24] Churchill MR, DeBoer BG (1975). Molecules with an M_4_X_4_ core. VII. Crystal and molecular structure of tetrameric triethylphosphinesilver(I) iodide. Inorg. Chem..

[25] Liu L (2016). Structure-dependent mechanochromism of two Ag(I) imidazolate chains. CrystEngComm.

[26] Rajput G, Yadav MK, Drew MGB, Singh N (2015). Impact of Ligand Framework on the Crystal Structures and Luminescent Properties of Cu(I) and Ag(I) Clusters and a Coordination Polymer Derived from Thiolate/Iodide/dppm Ligands. Inorg. Chem..

[27] Wan X-Y (2015). Structural variability, unusual thermochromic luminescence and nitrobenzene sensing properties of five Zn(II) coordination polymers assembled from a terphenyl-hexacarboxylate ligand. CrystEngComm.

[28] Perrucha S (2011). Thermochromic Luminescence of Copper Iodide Clusters: The Case of Phosphine Ligands. Inorg. Chem..

[29] Yersin H, Leitl MJ, Czerwieniec R (2014). In TADF for singlet harvesting: next generation OLED materials based on brightly green and blue emitting Cu(I) and Ag(I) compounds. Proc. Of SPIE.

[30] Kwon E, Kim J, Lee KY, Kim TH (2017). Non-Phase-Transition Luminescence Mechanochromism of a Copper(I) Coordination Polymer. Inorg. Chem..

[31] Crenshaw BR, Weder C (2003). Deformation-Induced Color Changes in Melt-Processed Photoluminescent Polymer Blends. Chem. Mater..

[32] Yoon S-J (2010). Multistimuli Two-Color Luminescence Switching via Different Slip-Stacking of Highly Fluorescent Molecular Sheets. J. Am. Chem. Soc..

[33] Wen T, Zhang D-X, Liu J, Lin R, Zhang J (2013). A multifunctional helical Cu(I) coordination polymer with mechanochromic, sensing and photocatalytic properties. Chem. Commun..

[34] Wen T, Zhou X-P, Zhang D-X, Li D (2014). Luminescent Mechanochromic Porous Coordination Polymers. Chem. Eur. J..

[35] Lavrenova A (2017). Mechano- and Thermoresponsive Photoluminescent Supramolecular Polymer. J. Am. Chem. Soc..

[36] Benito Q (2015). Pressure Control of Cuprophilic Interactions in a Luminescent Mechanochromic Copper Cluster. Inorg. Chem..

[37] Hayashi T (2015). Vapochromic Luminescence and Flexibility Control of Porous Coordination Polymers by Substitution of Luminescent Multinuclear Cu(I) Cluster Nodes. Inorg. Chem..

[38] Maderlehner S, Leitl MJ, Yersin H, Pfitzner A (2015). Halocuprate(I) zigzag chain structures with N-methylated DABCO cations-bright metal-centered luminescence and thermally activated color shifts. Dalton Trans..

[39] Kobayashi A, Arata R, Ogawa T, Yoshida M, Kato M (2017). Effect of Water Coordination on Luminescent Properties of Pyrazine-Bridged Dinuclear Cu(I) Complexes. Inorg. Chem..

[40] Chen X-L (2016). A strongly greenish-blue-emitting Cu_4_Cl_4_ cluster with an efficient spin-orbit coupling (SOC): fast phosphorescence versus thermally activated delayed fluorescence. Chem. Commun..

[41] Shan X-C (2013). Using cuprophilicity as a multi-responsive chromophore switching color in response to temperature, mechanical force and solvent vapors. J. Mater. Chem. C.

[42] Shen X-Y (2013). Effects of Substitution with Donor−Acceptor Groups on the Properties of Tetraphenylethene Trimer: Aggregation-Induced Emission, Solvatochromism, and Mechanochromism. J. Phys. Chem. C.

