# Supplementary material for: Observation of Contrary Thermo-responsive Trend for Single Crystal and Powder Samples in Mechano-, Thermo- and Solvato-responsive Luminescent Cubane [Ag4I4L4] Cluster
Source: Sci Rep. 2017 Oct 12;7:13058. doi: 10.1038/s41598-017-11974-8 (PMC5638816; doi:10.1038/s41598-017-11974-8)
Supplement: Supplementary file 1 — Supporting Information [file 41598_2017_11974_MOESM1_ESM.pdf]

Supporting Information

Observation of Contrary Thermo-responsive Trend for Single Crystal and Powder Samples in Mechano-, Thermo- and Solvato-responsive Luminescent Cubane [$Ag_4I_4L_4$] Cluster

Shi-Li Li,[†] Min Han,[†] Bin Wu,[†] Jie Wang,[†] Fu-Qiang Zhang,[†]
Xian-Ming Zhang[†‡]*

[†]*Key Laboratory of Magnetic Molecules & Magnetic Information Materials Ministry of Education, The School of Chemistry& Material Science, Shanxi Normal University. Linfen, Shanxi 041004, P. R. China*

[‡]*Institute of Crystalline Materials, Shanxi University, Taiyuan, Shanxi 030006, P. R. China*

Contents

S1. The Tables in Supporting Information 3

S2. The Figures in Supporting Information 5

Table S1. Selected Bonds Lengths (Å) and Angles (°) of **1** at 293 K.

Ag(1)-P(1)	2.4600(3)	Ag(2)-P(2)	2.450(4)
Ag(1)-I(2)	2.8782(11)	Ag(2)-I(2b)	2.9311(9)
Ag(1)-I(2a)	2.9122(11)	Ag(2)-I(2)	2.9310(9)
Ag(1)-I(1)	2.9271(12)	Ag(2)-I(2a)	2.9311(9)
Ag(1)-Ag(1a)	3.3762(15)	Ag(1)-Ag(1b)	3.3764(15)
P(1)-Ag(1)-I(2)	118.73(7)	P(2)-Ag(2)-I(2b)	120.27(3)
P(1)-Ag(1)-I(2a)	112.09(7)	P(2)-Ag(2)-I(2)	120.26 (3)
I(2)-Ag(1)-I(2a)	98.44(4)	I(2b)-Ag(2)-I(2)	96.83(3)
P(1)-Ag(1)-I(1)	111.74(7)	P(2)-Ag(2)-I(2a)	120.27(3)
I(2)-Ag(1)-I(1)	107.74(3)	I(2b)-Ag(2)-I(2a)	96.83(3)
I(2a)-Ag(1)-I(1)	106.83(3)	I(2)-Ag(2)-I(2a)	96.83(3)

Symmetry Codes: a) -z+1, x-1/2, -y+1/2 b) y+1/2, -z+1/2, -x+1

Table S2. π···π interaction in compound **1**.

The intermolecular offset π···π interaction	
**R(i) → R(i)**	Distance between the two Cg centroids (Å)
**R(1) → R(1)**	3.900(8)

R(i) denotes the centroids of the ith rings, R(1) C15-C16-C17-C18-C19-C20. Symmetry codes:(i) 2-X, 1/2-Y, Z.

Table S3. Emission lifetime for single crystal **1** at variable temperatures.

**T/K**	**λ_1/η (μs)**	**λ_2/η(μs)**
**5**	440/97.4	466/90.5
**10**	440/92.5	466/86.9
**50**	440/72.9	466/51.4

**75**	440/64.3	466/50.3
**100**	440/50.1	466/32.8
**150**	440/33.6	466/24.5
**200**	447/21.7	469/23.6
**250**	448/11.8	473/9.4
**300**	448/0.8	476/1.3

Table S4. Emission lifetime for ground **1** at variable temperatures.

**T/K**	**λ_1/η(μs)**	**λ_2/η(μs)**
**5**	435/168.5	465/175.6
**10**	435/136.6	465/166.5
**50**	435/93.8	465/90.3
**75**	435/58.6.7	465/57.9
**100**	435/55.6	465/56.8
**150**	430/34.9	471/32.6
**200**	430/11.9	468/12.8
**250**	424/3.4	468/5.1
**300**	418/0.4	468/0.6

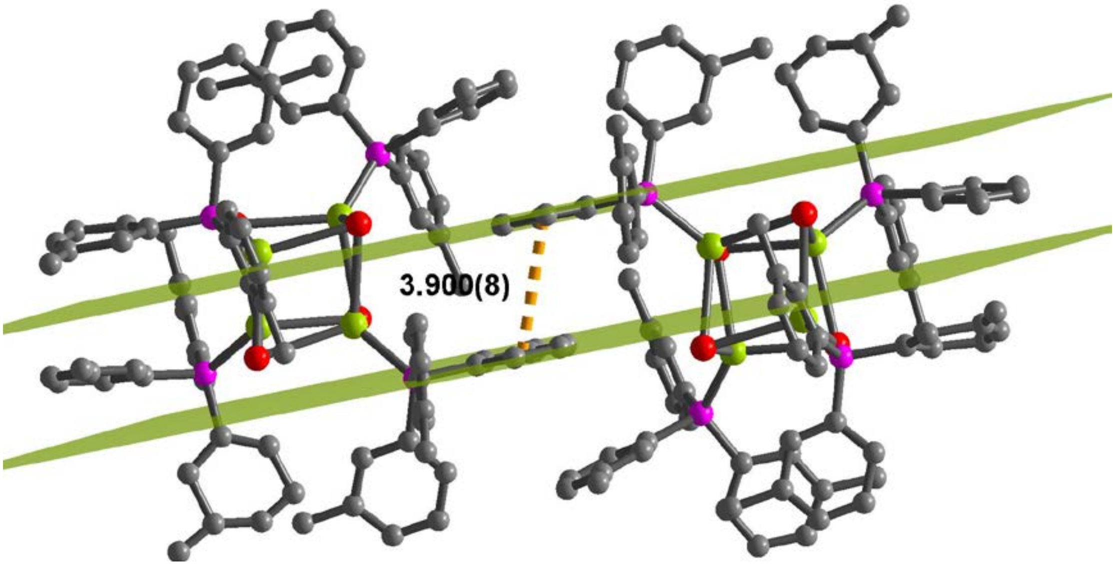

Figure S1. π···π interactions among the ligands of compound **1**.

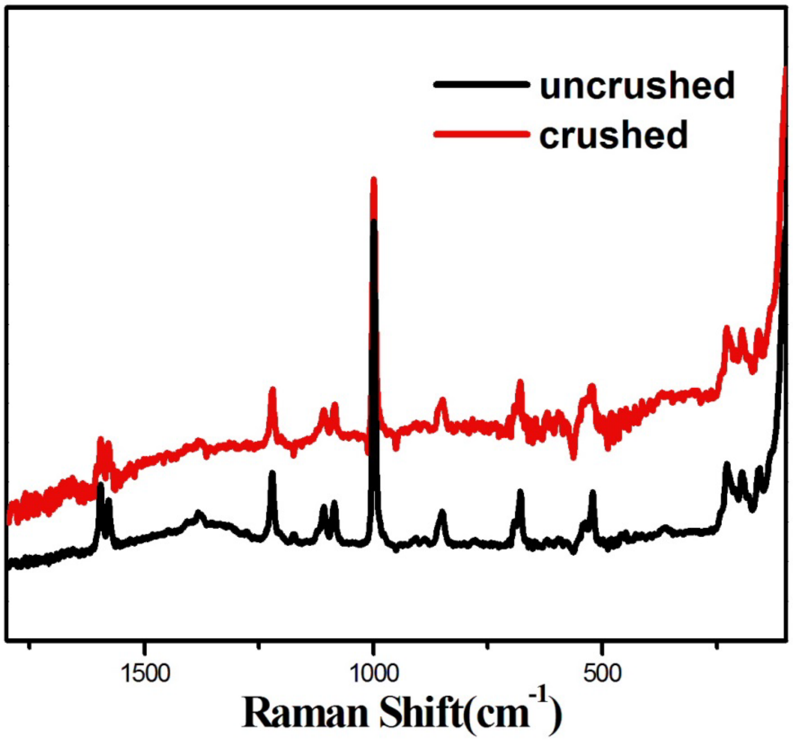

Figure S2. Raman spectra of uncrushed and crushed sample **1**.

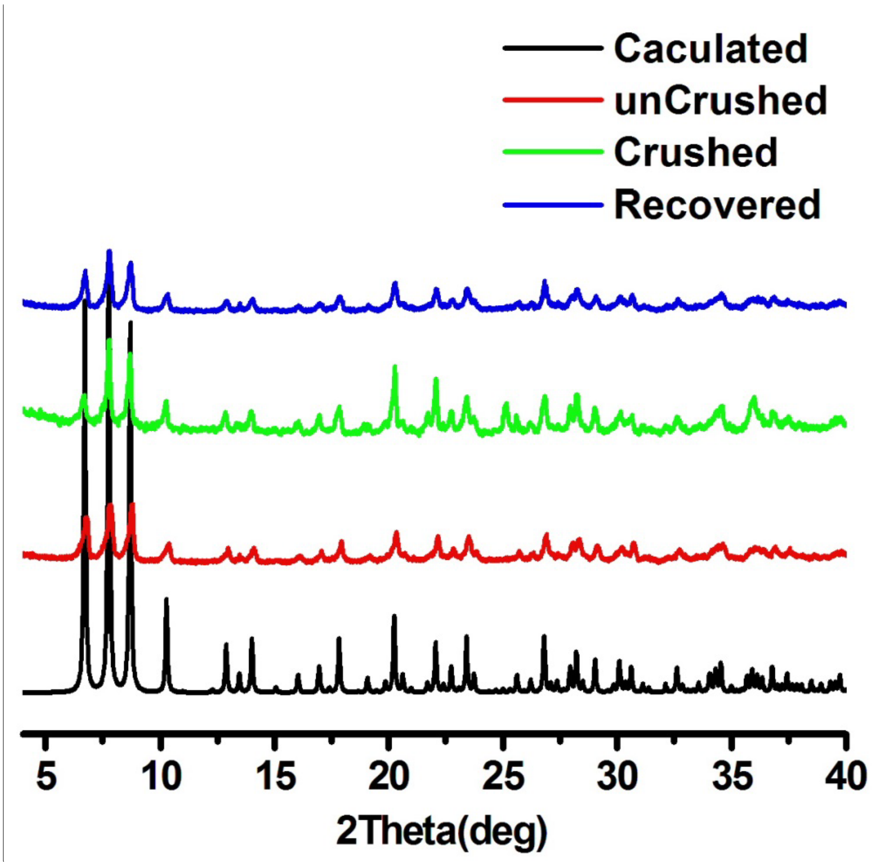

Figure S3. PXRD curves of **1** in the different solid state.

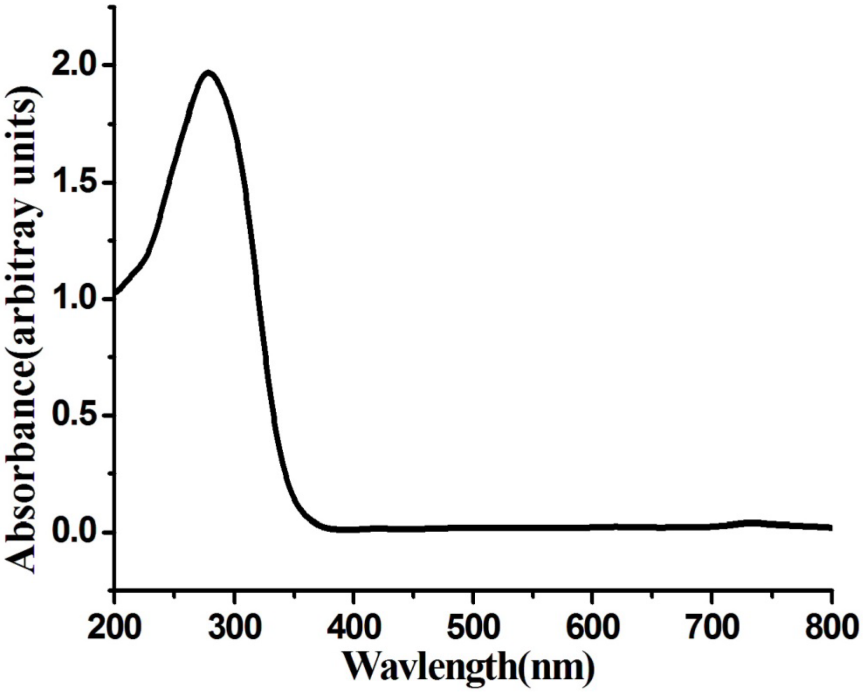

Figure S4. UV-vis Spectra of powder sample **1.**

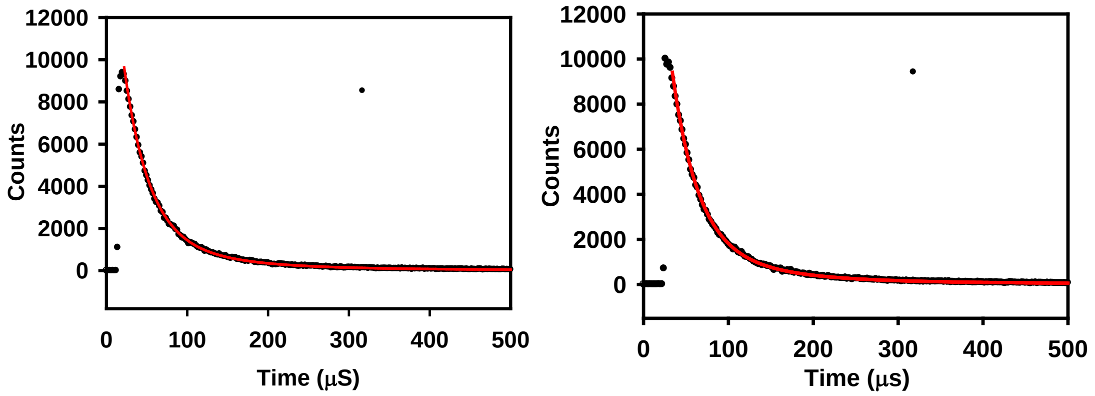

Figure S5. Emission lifetime curves and corresponding fits of single crystal **1** for HE band (right) and LE band (left) at 5 K.

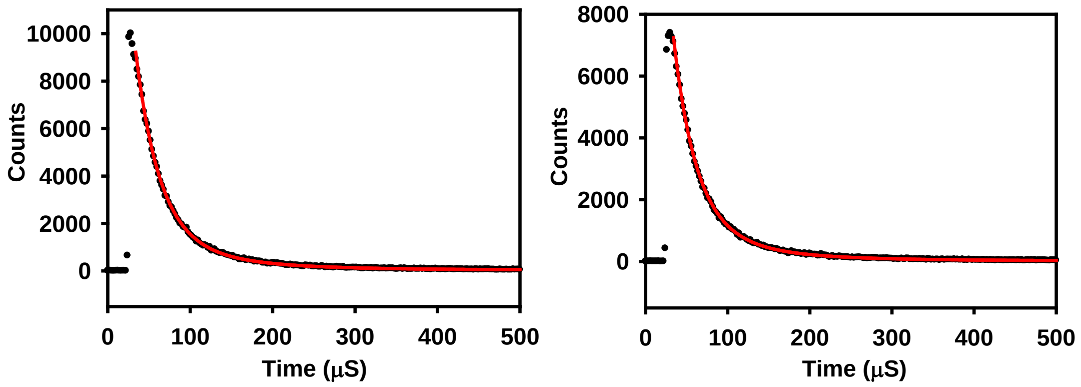

Figure S6. Emission lifetime curves and corresponding fits of single crystal **1** for HE band (right) and LE band (left) at 10 K.

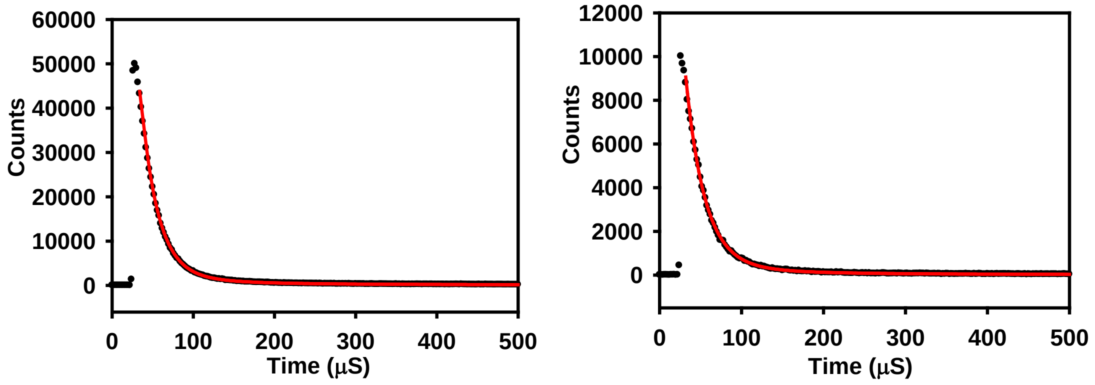

Figure S7. Emission lifetime curves and corresponding fits of single crystal **1** for HE band (right) and LE band (left) at 50 K.

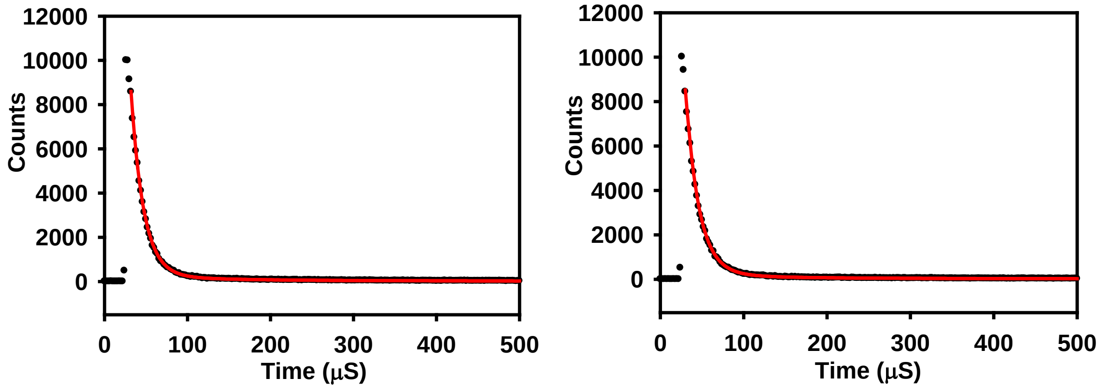

Figure S8. Emission lifetime curves and corresponding fits of single crystal **1** for HE band (right) and LE band (left) at 75 K.

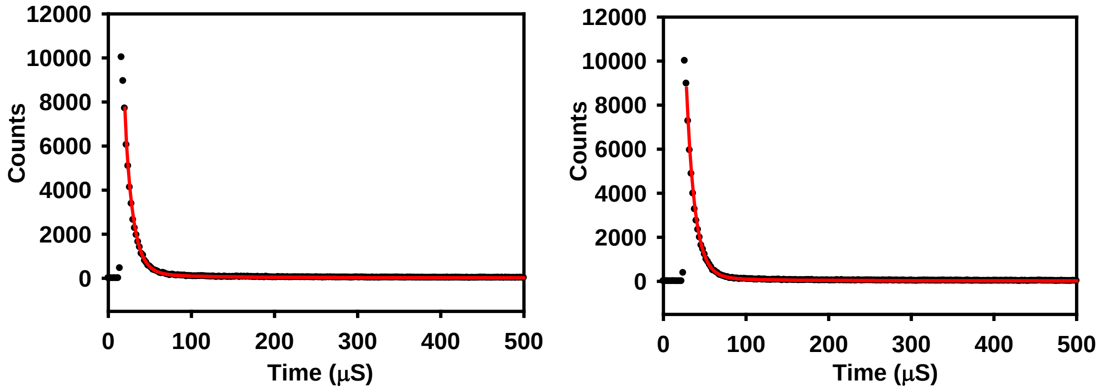

Figure S9. Emission lifetime curves and corresponding fits of single crystal **1** for HE band (right) and LE band (left) at 100 K.

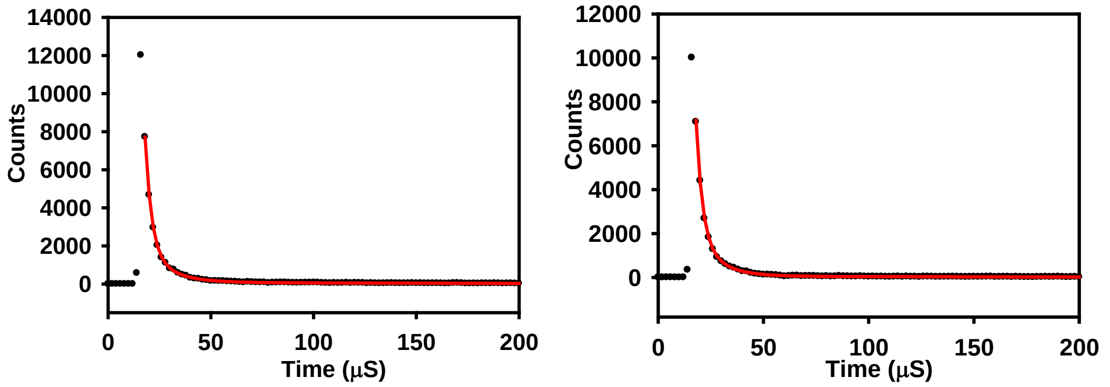

Figure S10. Emission lifetime curves and corresponding fits of single crystal **1** for HE band (right) and LE band (left) at 150 K.

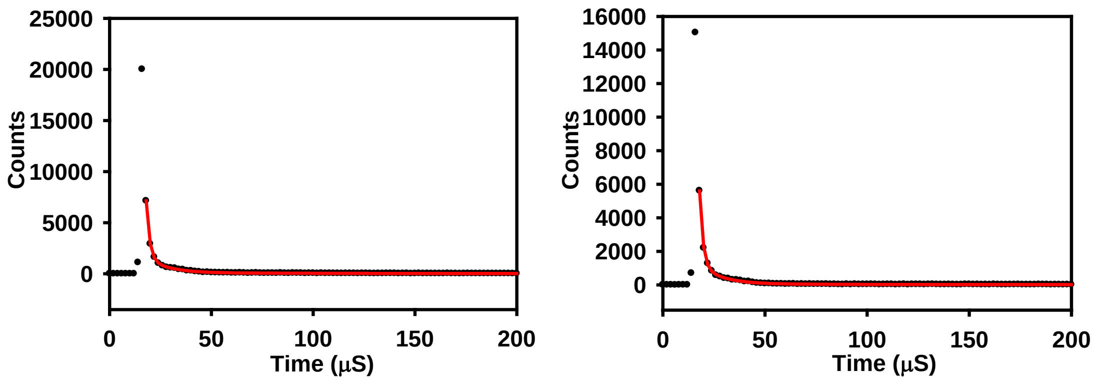

Figure S11. Emission lifetime curves and corresponding fits of single crystal **1** for HE band (right) and LE band (left) at 200 K.

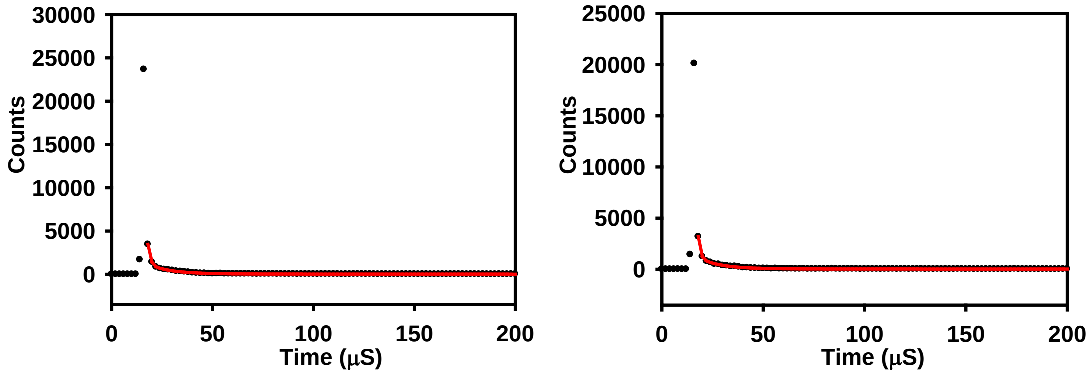

Figure S12. Emission lifetime curves and corresponding fits of single crystal **1** for HE band (right) and LE band (left) at 250 K.

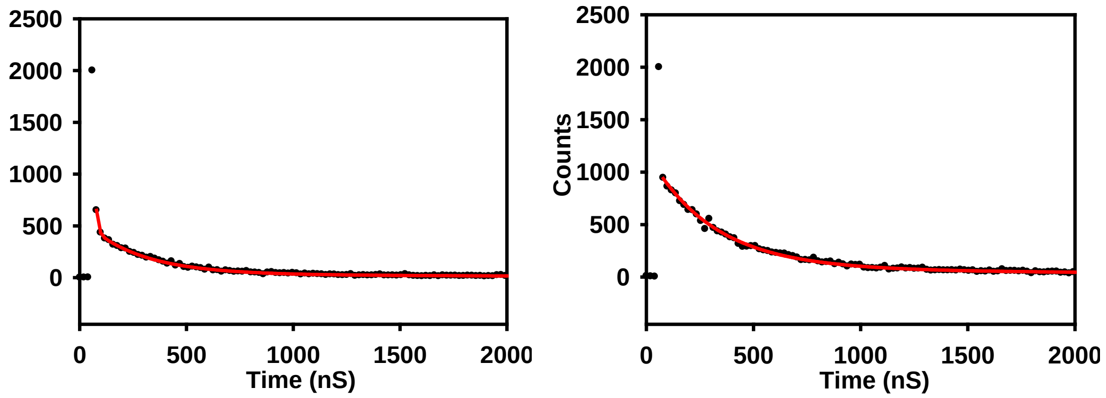

Figure S13. Emission lifetime curves and corresponding fits of single crystal **1** for HE band (right) and LE band (left) at 300 K.

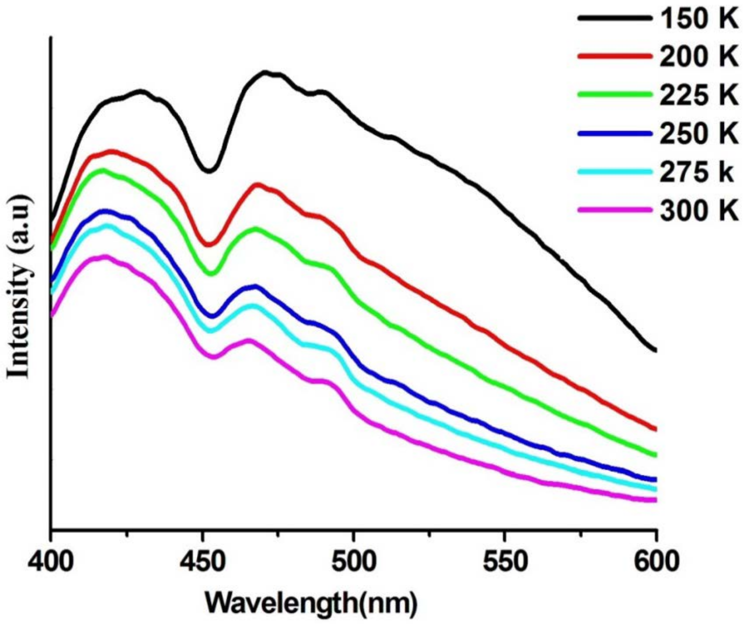

Figure S14. Temperature-dependent emission spectra of compound **1** in ground powder state from 150 to 300 K.

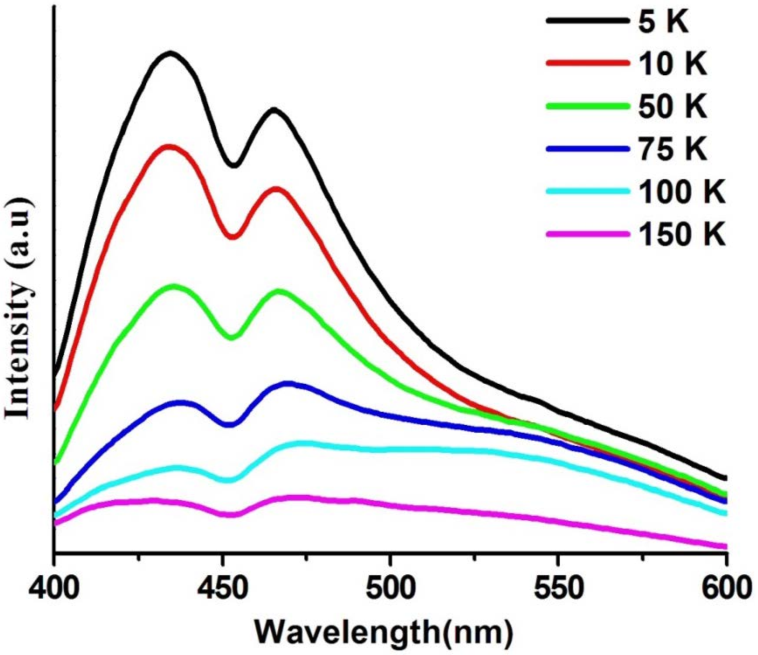

Figure S15. Temperature-dependent emission spectra of compound **1** in ground powder state from 5 to 150 K.

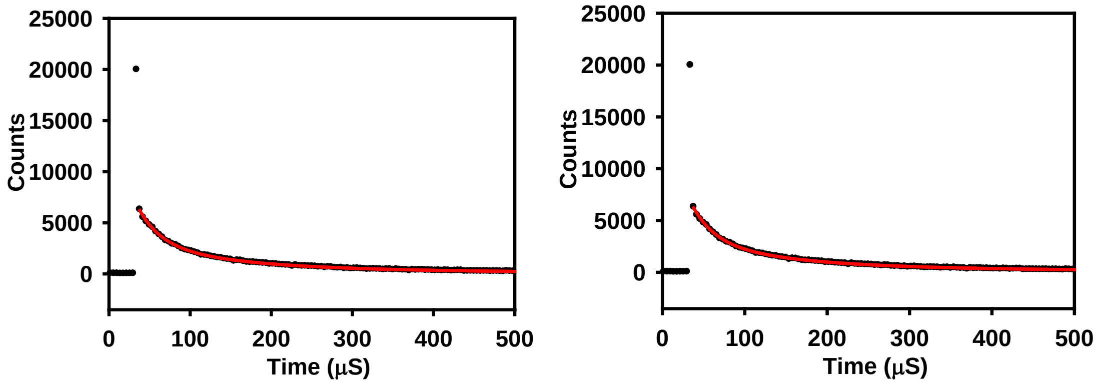

Figure S16. Emission lifetime curves and corresponding fits of powder sample **1** for HE band (right) and LE band (left) at 5 K.

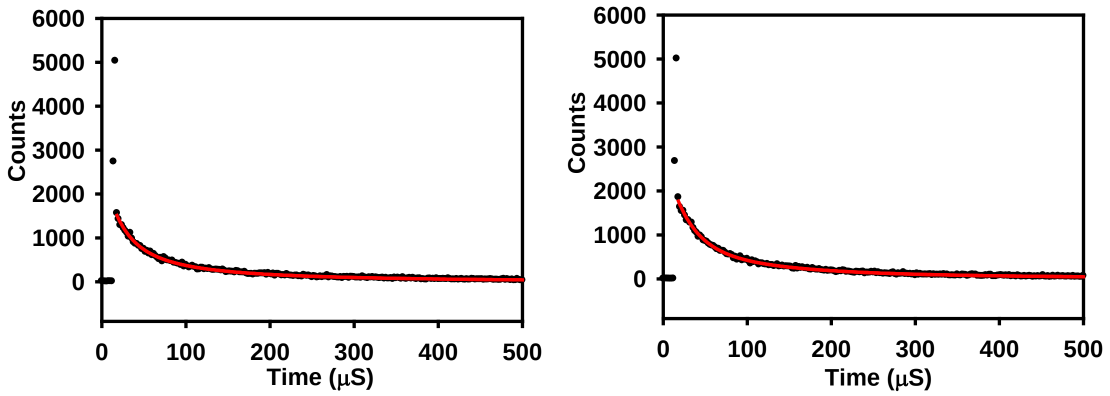

Figure S17. Emission lifetime curves and corresponding fits of powder sample **1** for HE band (right) and LE band (left) at 10 K.

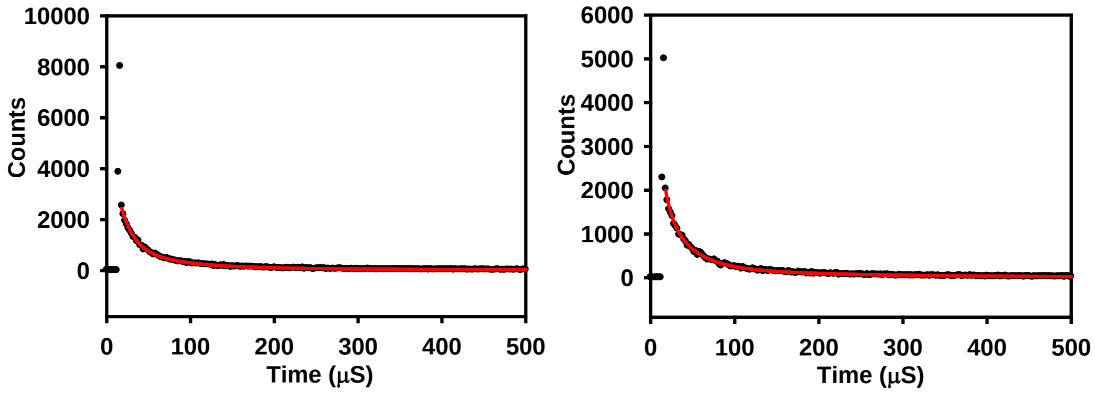

Figure S18. Emission lifetime curves and corresponding fits of powder sample **1** for HE band (right) and LE band (left) at 50 K.

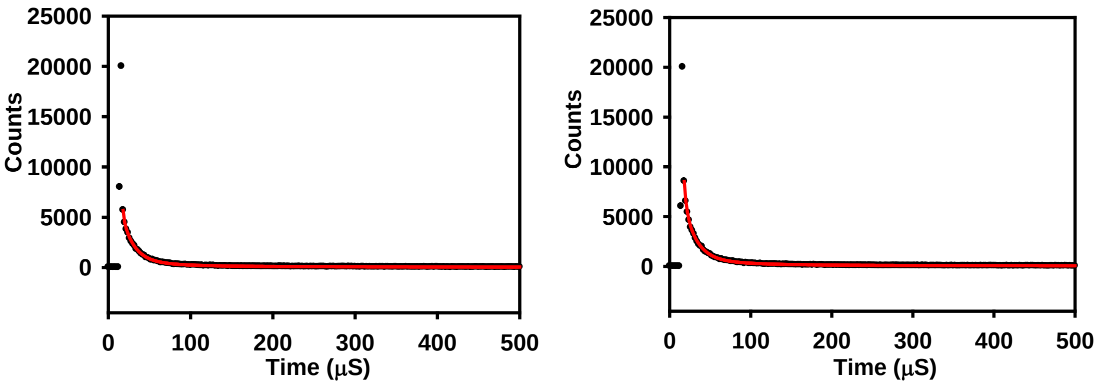

Figure S19. Emission lifetime curves and corresponding fits of powder sample **1** for HE band (right) and LE band (left) at 75 K.

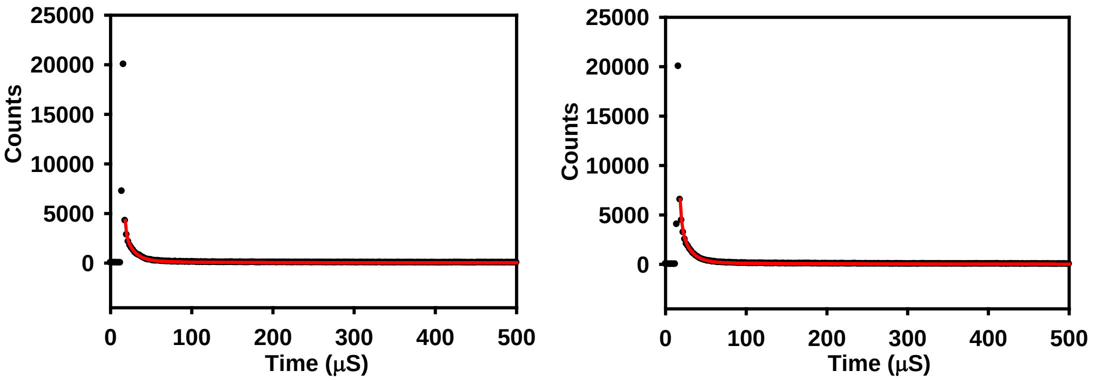

Figure S20. Emission lifetime curves and corresponding fits of powder sample **1** for HE band (right) and LE band (left) at 100 K.

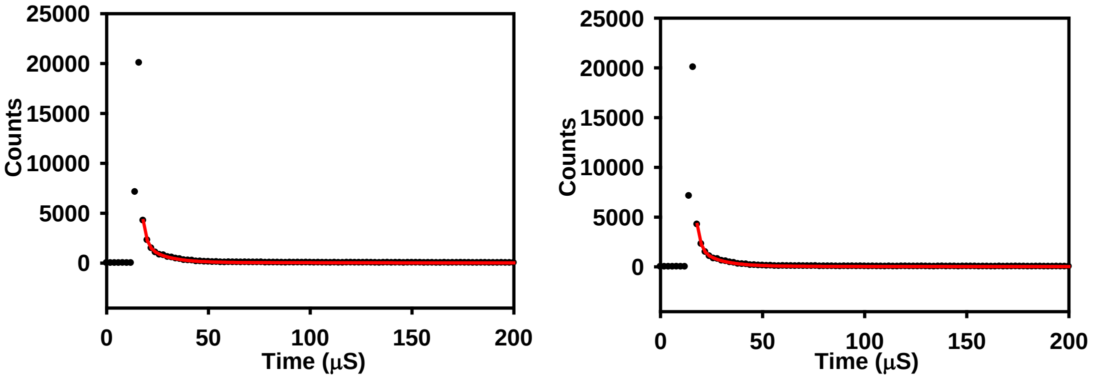

Figure S21. Emission lifetime curves and corresponding fits of powder sample **1** for HE band (right) and LE band (left) at 150 K.

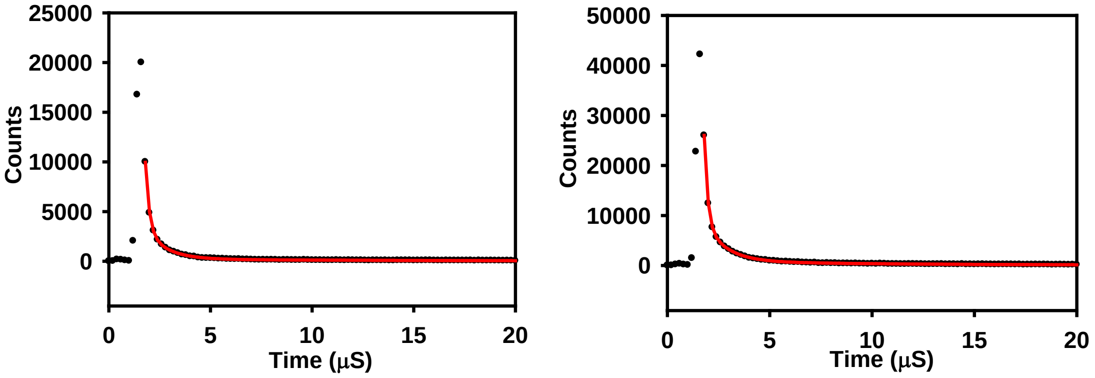

Figure S22. Emission lifetime curves and corresponding fits of powder sample **1** for HE band (right) and LE band (left) at 200 K.

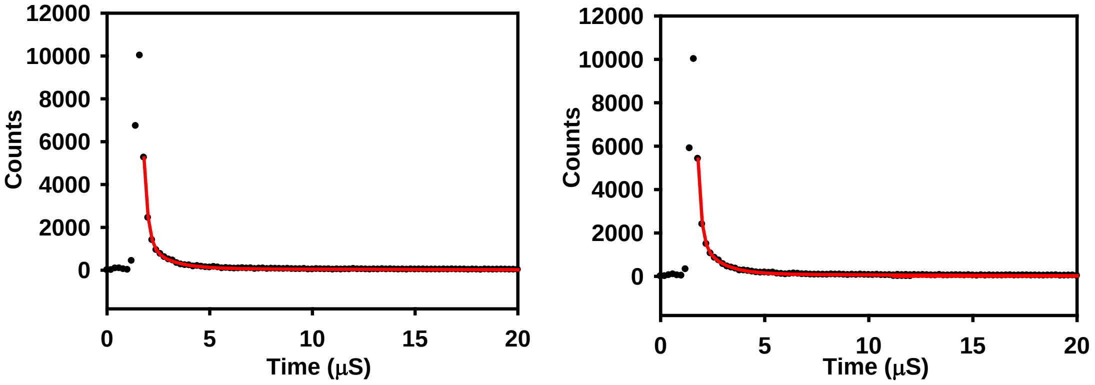

Figure S23. Emission lifetime curves and corresponding fits of powder sample **1** for HE band (right) and LE band (left) at 250 K.

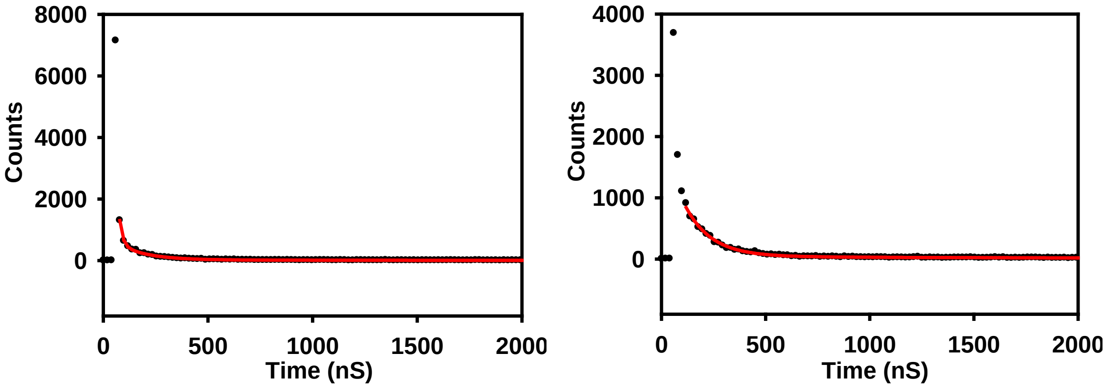

Figure S24. Emission lifetime curves and corresponding fits of powder sample **1** for HE band (right) and LE band (left) at 300 K.

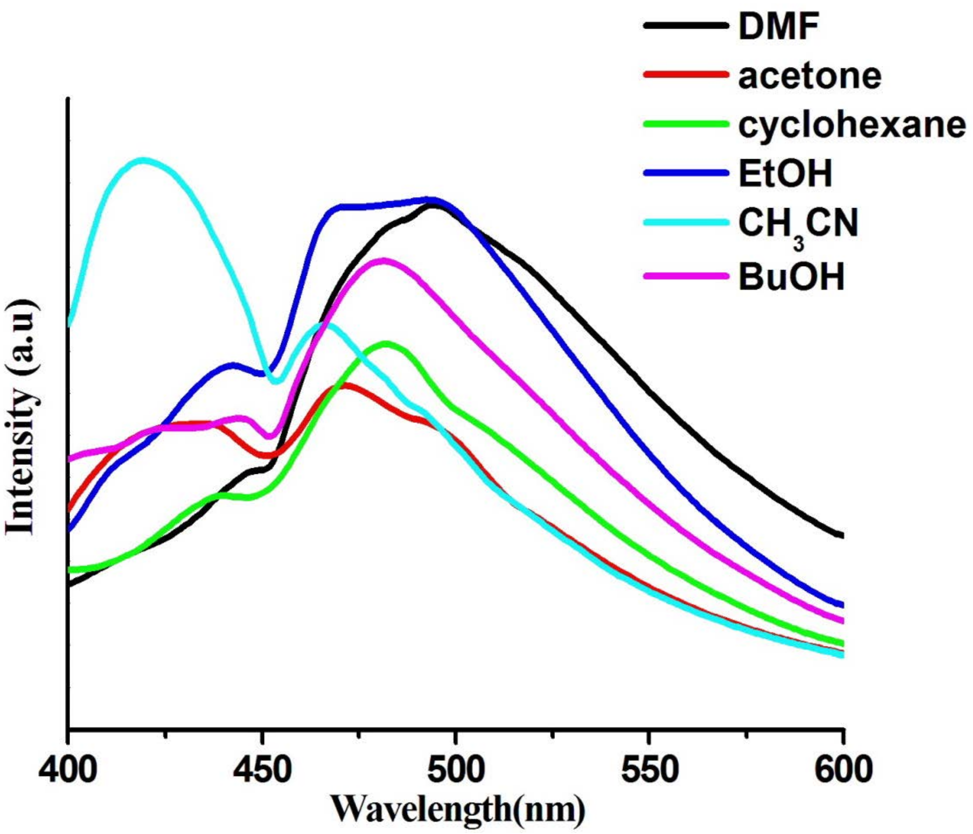

Figure S25. Emission spectra of **1** in different solvents excited by 340 nm.

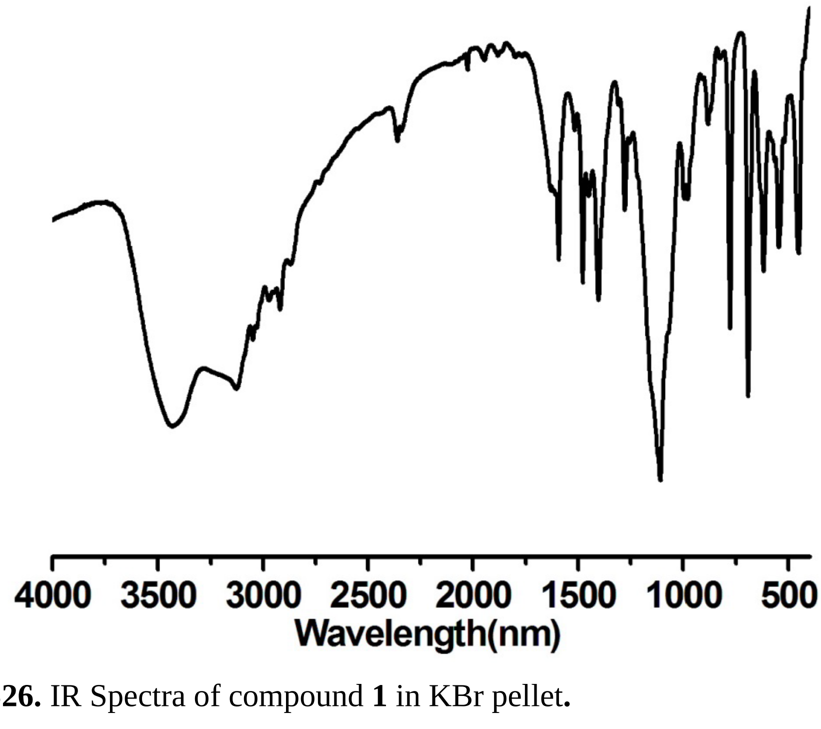

Figure S26. IR Spectra of compound **1** in KBr pellet**.**

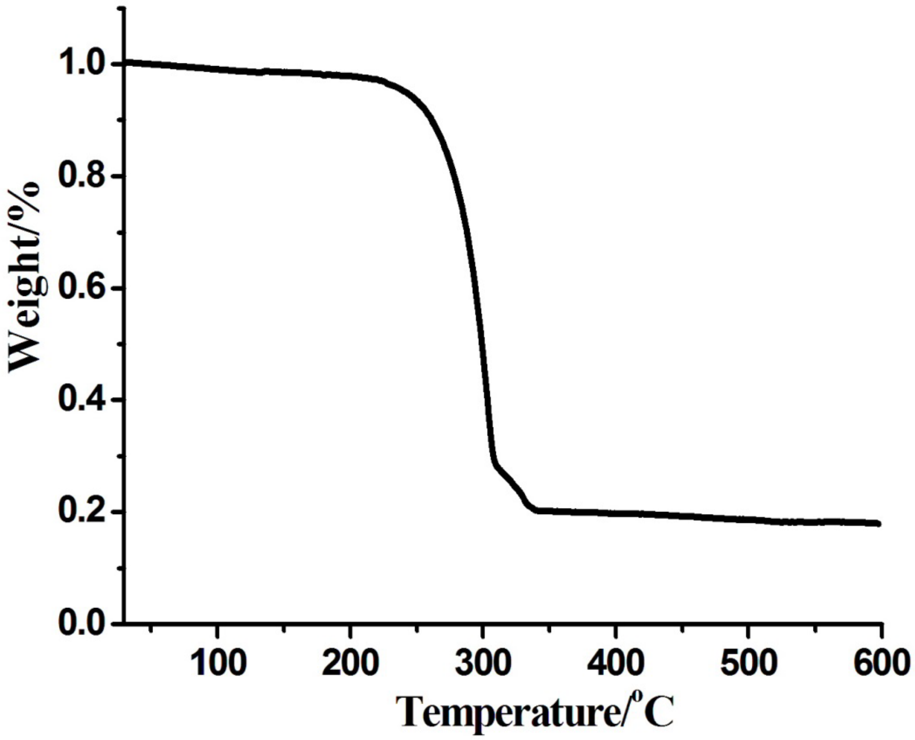

Figure S27. The TG plots for **1** under nitrogen atmosphere at heating rate of 10 °C/min.
