# Supplementary material for: Observation of Contrary Thermo-responsive Trend for Single Crystal and Powder Samples in Mechano-, Thermo- and Solvato-responsive Luminescent Cubane [Ag4I4L4] Cluster
Source: Sci Rep. 2017 Oct 12;7:13058. doi: 10.1038/s41598-017-11974-8 (PMC5638816; doi:10.1038/s41598-017-11974-8)

checkCIF/PLATON report

Structure factors have been supplied for datablock(s) a

THIS REPORT IS FOR GUIDANCE ONLY. IF USED AS PART OF A REVIEW PROCEDURE FOR PUBLICATION, IT SHOULD NOT REPLACE THE EXPERTISE OF AN EXPERIENCED CRYSTALLOGRAPHIC REFEREE.

No syntax errors found. CIF dictionary Interpreting this report

Datablock: a

```
Bond precision:  C-C = 0.0204 A          Wavelength=0.71073

Cell:            a=32.2143(7)      b=32.2143(7)      c=32.2143(7)
                 alpha=90          beta=90           gamma=90
Temperature:     293 K

                Calculated                  Reported
Volume          33431(2)                    33431(2)
Space group     I -4 3 d                    I -4 3 d
Hall group      I -4bd 2c 3                 I -4bd 2c 3
Moiety formula  C84 H87 Ag4 I4 P4           C84 H87 Ag4 I4 P4
Sum formula     C84 H87 Ag4 I4 P4           C84 H87 Ag4 I4 P4
Mr              2159.49                     2159.49
Dx,g cm-3       1.716                       1.716
Z               16                          16
Mu (mm-1)       2.518                       2.518
F000            16816.0                     16816.0
F000'           16739.15
h,k,lmax        40,40,40                    40,40,40
Nref            5695[ 3007]                 5704
Tmin,Tmax       0.586,0.777                 0.659,1.000
Tmin'           0.514

Correction method= # Reported T Limits: Tmin=0.659 Tmax=1.000
AbsCorr = MULTI-SCAN

Data completeness= 1.90/1.00      Theta(max)= 26.359

R(reflections)= 0.0358( 4471)     wR2(reflections)= 0.0958( 5704)

S = 1.031                    Npar= 290
```

The following ALERTS were generated. Each ALERT has the format
test-name_ALERT_alert-type_alert-level.
Click on the hyperlinks for more details of the test.

Alert level B

```
PLAT230_ALERT_2_B Hirshfeld Test Diff for    C9     --  C10     ..        7.4 s.u.
PLAT234_ALERT_4_B Large Hirshfeld Difference C10    --  C14     ..       0.30 Ang.
PLAT234_ALERT_4_B Large Hirshfeld Difference C11    --  C12     ..       0.28 Ang.
PLAT241_ALERT_2_B High   'MainMol' Ueq as Compared to Neighbors of        C12 Check
PLAT342_ALERT_3_B Low Bond Precision on  C-C Bonds ...............    0.02043 Ang.
PLAT362_ALERT_2_B Short   C(sp3)-C(sp2) Bond  C10    -   C14    ..       1.22 Ang.
```

Alert level C

```
PLAT220_ALERT_2_C Non-Solvent Resd 1   C   Ueq(max)/Ueq(min) Range        3.4 Ratio
PLAT222_ALERT_3_C Non-Solvent Resd 1   H Uiso(max)/Uiso(min) Range        4.4 Ratio
PLAT230_ALERT_2_C Hirshfeld Test Diff for    C12    --  C13     ..        6.5 s.u.
PLAT234_ALERT_4_C Large Hirshfeld Difference C8     --  C9      ..       0.22 Ang.
PLAT234_ALERT_4_C Large Hirshfeld Difference C10    --  C11     ..       0.21 Ang.
PLAT234_ALERT_4_C Large Hirshfeld Difference C24    --  C25     ..       0.23 Ang.
PLAT241_ALERT_2_C High   'MainMol' Ueq as Compared to Neighbors of        Ag1 Check
PLAT241_ALERT_2_C High   'MainMol' Ueq as Compared to Neighbors of         C9 Check
PLAT241_ALERT_2_C High   'MainMol' Ueq as Compared to Neighbors of        C13 Check
PLAT241_ALERT_2_C High   'MainMol' Ueq as Compared to Neighbors of        C18 Check
PLAT241_ALERT_2_C High   'MainMol' Ueq as Compared to Neighbors of        C25 Check
PLAT242_ALERT_2_C Low    'MainMol' Ueq as Compared to Neighbors of         C8 Check
PLAT242_ALERT_2_C Low    'MainMol' Ueq as Compared to Neighbors of        C10 Check
PLAT242_ALERT_2_C Low    'MainMol' Ueq as Compared to Neighbors of        C11 Check
PLAT242_ALERT_2_C Low    'MainMol' Ueq as Compared to Neighbors of        C17 Check
PLAT242_ALERT_2_C Low    'MainMol' Ueq as Compared to Neighbors of        C24 Check
PLAT362_ALERT_2_C Short   C(sp3)-C(sp2) Bond  C8     -   C9     ..       1.37 Ang.
PLAT363_ALERT_2_C Long    C(sp3)-C(sp2) Bond  C9     -   C10    ..       1.64 Ang.
PLAT368_ALERT_2_C Short   C(sp2)-C(sp2) Bond  C11    -   C12    ..       1.21 Ang.
PLAT411_ALERT_2_C Short Inter H...H Contact   H9B    ..  H26     .       2.06 Ang.
PLAT601_ALERT_2_C Structure Contains Solvent Accessible VOIDS of .         46 Ang3
PLAT978_ALERT_2_C Number C-C Bonds with Positive Residual Density.          0 Note
```

Alert level G

```
PLAT003_ALERT_2_G Number of Uiso or Uij Restrained non-H Atoms ...          1 Report
PLAT083_ALERT_2_G SHELXL Second Parameter in WGHT  Unusually Large      59.76 Why ?
PLAT093_ALERT_1_G No s.u.'s on H-positions, Refinement Reported as      mixed Check
PLAT186_ALERT_4_G The CIF-Embedded .res File Contains ISOR Records          1 Report
PLAT199_ALERT_1_G Reported _cell_measurement_temperature ..... (K)        293 Check
PLAT200_ALERT_1_G Reported   _diffrn_ambient_temperature ..... (K)        293 Check
PLAT232_ALERT_2_G Hirshfeld Test Diff (M-X)  I1     --  Ag1     ..       27.5 s.u.
PLAT232_ALERT_2_G Hirshfeld Test Diff (M-X)  I2     --  Ag1     ..       17.3 s.u.
PLAT232_ALERT_2_G Hirshfeld Test Diff (M-X)  I2     --  Ag2     ..       18.2 s.u.
PLAT232_ALERT_2_G Hirshfeld Test Diff (M-X)  I2     --  Ag1_b   ..       38.0 s.u.
PLAT232_ALERT_2_G Hirshfeld Test Diff (M-X)  Ag2    --  P2      ..        8.3 s.u.
PLAT380_ALERT_4_G Incorrectly? Oriented X(sp2)-Methyl Moiety .....         C7 Check
PLAT380_ALERT_4_G Incorrectly? Oriented X(sp2)-Methyl Moiety .....        C14 Check
PLAT380_ALERT_4_G Incorrectly? Oriented X(sp2)-Methyl Moiety .....        C21 Check
PLAT380_ALERT_4_G Incorrectly? Oriented X(sp2)-Methyl Moiety .....        C28 Check
PLAT860_ALERT_3_G Number of Least-Squares Restraints .............          6 Note
PLAT996_ALERT_1_G Non-Standard SHELXL LIST 4 Style FCF Supplied ..          ! Check
```

0 **ALERT level A** = Most likely a serious problem - resolve or explain
6 **ALERT level B** = A potentially serious problem, consider carefully
22 **ALERT level C** = Check. Ensure it is not caused by an omission or oversight
17 **ALERT level G** = General information/check it is not something unexpected

4 ALERT type 1 CIF construction/syntax error, inconsistent or missing data

```
28 ALERT type 2 Indicator that the structure model may be wrong or deficient
 3 ALERT type 3 Indicator that the structure quality may be low
10 ALERT type 4 Improvement, methodology, query or suggestion
 0 ALERT type 5 Informative message, check
```

It is advisable to attempt to resolve as many as possible of the alerts in all categories. Often the minor alerts point to easily fixed oversights, errors and omissions in your CIF or refinement strategy, so attention to these fine details can be worthwhile. In order to resolve some of the more serious problems it may be necessary to carry out additional measurements or structure refinements. However, the purpose of your study may justify the reported deviations and the more serious of these should normally be commented upon in the discussion or experimental section of a paper or in the "special_details" fields of the CIF. checkCIF was carefully designed to identify outliers and unusual parameters, but every test has its limitations and alerts that are not important in a particular case may appear. Conversely, the absence of alerts does not guarantee there are no aspects of the results needing attention. It is up to the individual to critically assess their own results and, if necessary, seek expert advice.

Publication of your CIF in IUCr journals

A basic structural check has been run on your CIF. These basic checks will be run on all CIFs submitted for publication in IUCr journals (*Acta Crystallographica*, *Journal of Applied Crystallography*, *Journal of Synchrotron Radiation*); however, if you intend to submit to *Acta Crystallographica Section C* or *E* or *IUCrData*, you should make sure that full publication checks are run on the final version of your CIF prior to submission.

Publication of your CIF in other journals

Please refer to the *Notes for Authors* of the relevant journal for any special instructions relating to CIF submission.

PLATON version of 27/03/2017; check.def file version of 24/03/2017

Datablock a - ellipsoid plot

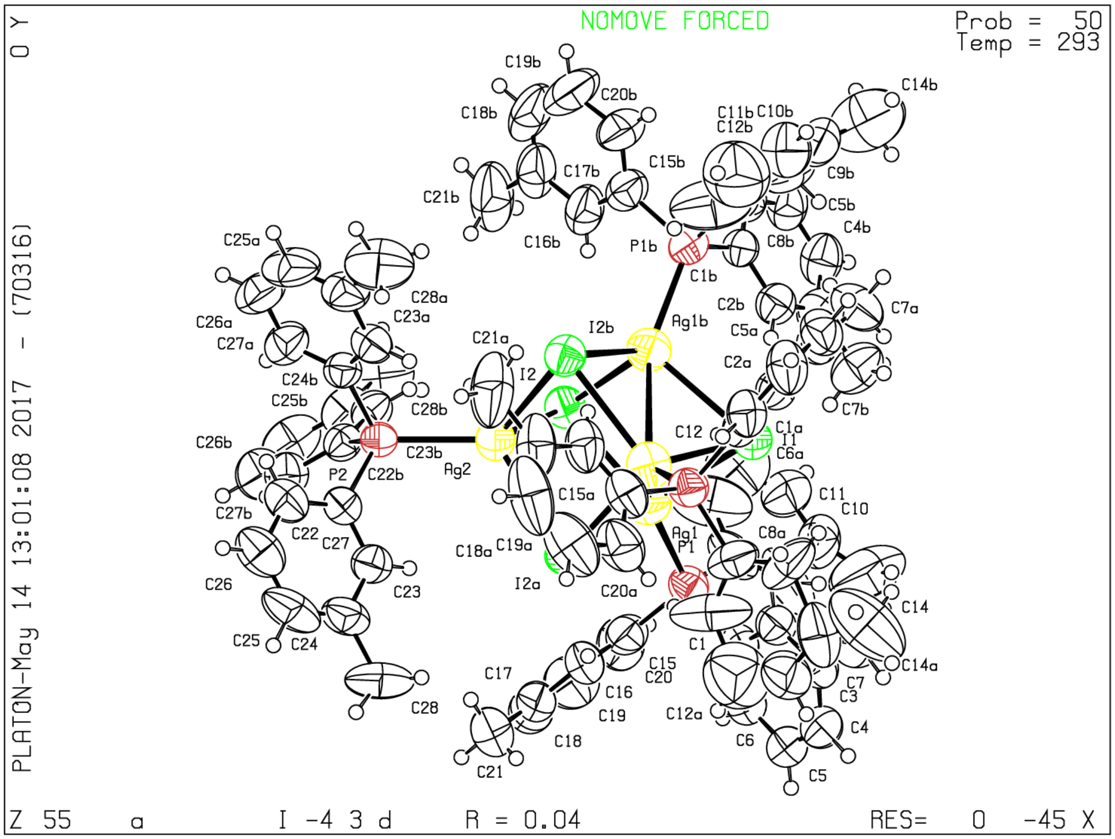

Supplement: Supplementary file 3 — the checkCIF PDF [file 41598_2017_11974_MOESM3_ESM.pdf]
